# Cross‐validation of equations to predict whole‐body sweat sodium concentration from regional measures during exercise

**DOI:** 10.14814/phy2.14524

**Published:** 2020-08-03

**Authors:** Lindsay B. Baker, Ryan P. Nuccio, Adam J. Reimel, Shyretha D. Brown, Corey T. Ungaro, Peter John D. De Chavez, Kelly A. Barnes

**Affiliations:** ^1^ Gatorade Sports Science Institute PepsiCo R&D Life Sciences Barrington IL USA; ^2^ PepsiCo R&D Barrington IL USA

**Keywords:** electrolytes, heat stress, sweating rate, tattoos

## Abstract

We have previously published equations to estimate whole‐body (WB) sweat sodium concentration ([Na^+^]) from regional (REG) measures; however, a cross‐validation is needed to corroborate the applicability of these prediction equations between studies. The purpose of this study was to determine the validity of published equations in predicting WB sweat [Na^+^] from REG measures when applied to a new data set. Forty‐nine participants (34 men, 15 women; 75 ± 12 kg) cycled for 90 min while WB sweat [Na^+^] was measured using the washdown technique. REG sweat [Na^+^] was measured from seven regions using absorbent patches (3M Tegaderm + Pad). Published equations were applied to REG sweat [Na^+^] to determine predicted WB sweat [Na^+^]. Bland–Altman analysis of mean bias (raw and predicted minus measured) and 95% limits of agreement (LOA) were used to compare raw (uncorrected) REG sweat [Na^+^] and predicted WB sweat [Na^+^] to measured WB sweat [Na^+^]. Mean bias (±95% LOA) between raw REG sweat [Na^+^] and measured WB sweat [Na^+^] was 10(±20), 0(±19), 9(±20), 22(±25), 23(±24), 0(±15), −4(±18) mmol/L for the dorsal forearm, ventral forearm, upper arm, chest, upper back, thigh, and calf, respectively. The mean bias (±95% LOA) between predicted WB sweat [Na^+^] and measured WB sweat [Na^+^] was 3(±14), 4(±12), 0(±14), 2(±17), −2(±16), 5(±13), 4(±15) mmol/L for the dorsal forearm, ventral forearm, upper arm, chest, upper back, thigh, and calf, respectively. Prediction equations improve the accuracy of estimating WB sweat [Na^+^] from REG and are therefore recommended for use when determining individualized sweat electrolyte losses.

## Graphical abstract

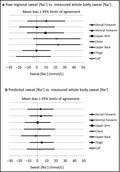

## 1 INTRODUCTION

Individualized sweat testing to determine sweat sodium concentration ([Na^+^]) is helpful in developing customized fluid/electrolyte replacement strategies for athletes (McDermott et al., 2017; Sawka et al., 2007). The regional absorbent sweat patch technique is a practical means to collect sweat during exercise for subsequent analysis of sweat [Na^+^] (Barnes et al., 2019). Although a convenient and commonly used method, regional sweat [Na^+^] is not representative of the entire body surface area (Shirreffs & Maughan, 1997). Therefore, previous studies have assessed the relation between regional and whole‐body sweat [Na^+^] and developed regression equations to predict whole‐body sweat [Na^+^] from regional measures (Baker, Stofan, Hamilton, & Horswill, 2009; Baker et al., 2018, 2019). These previous studies have reported the impact of various within‐subject (bilateral sides, day‐to‐day, exercise intensity) and between‐subject (sex) factors on the relation between regional and whole‐body sweat [Na^+^] (Baker et al., 2018, 2019). Based on these findings, the best regional sites for predicting whole‐body sweat [Na^+^] have also been proposed (e.g., forearm) (Baker et al., 2019).

Additional validation experiments are needed before whole‐body sweat [Na^+^] prediction models can be recommended for generalized use. For instance, it is unknown if the validity of the published regression equations holds true when applied to a new data set, such as via a cross‐validation analysis. Furthermore, as most previous studies have been conducted in a narrow range of environmental conditions (~30°C, 40%–45% relative humidity) (Baker et al., 2009, 2018, 2019), the validity of the regression equations outside of this range is unknown. While it is well‐established that differences in relative humidity can impact sweating rate (Candas, Libert, & Vogt, 1983; Collins & Weiner, 1962; Randall & Peiss, 1957), the impact on sweat electrolyte concentrations has not been investigated to date. Therefore, the primary objective of this study was to determine the validity of our published regression equations (Baker et al., 2018) in predicting whole‐body sweat [Na^+^] from regional measures when applied to a new data set acquired under similar experimental conditions. The secondary objective was to determine how a change in ambient humidity impacts the accuracy of whole‐body sweat [Na^+^] predictions.

It is also important to note that previous studies have included predominately non‐Hispanic, Caucasian athletes of moderate aerobic fitness status and with nontattooed skin at the sites of regional sweat collection (Baker et al., 2009, 2018, 2019). While standardization of such subject characteristics is commonly done in research to reduce the effect of between‐subject variability, it renders the ecological validity and generalizability of results ambiguous. Currently, the impact of race/ethnicity, fitness status, and tattoos on sweat electrolyte concentrations is equivocal, due in part to the limited research available. For instance, tattooed skin has been associated with significantly higher regional sweat [Na^+^] with pharmacologically induced sweating in one study (Luetkemeier, Hanisko, & Aho, 2017) but had no impact on regional sweat [Na^+^] in studies involving exercise‐induced sweating (Beliveau et al., 2020; Rogers, Irwin, McCartney, Cox, & Desbrow, 2019). If tattoos or other factors impact sweat [Na^+^] concentration at the regional level, this could in turn impact the relation between regional and whole‐body sweat [Na^+^]. Therefore, this study included a convenience sample of subjects with or without tattoos and of various race/ethnicities and fitness levels to gain preliminary insights regarding the impact of these factors on the accuracy of whole‐body sweat [Na^+^] predictions. In this manner, the generalizability of the prediction models can be determined for scenarios when environmental conditions and subject characteristics vary from that of the original studies.

## 2 MATERIALS AND METHODS

### 2.1 Subjects

Forty‐nine subjects (34 men, 15 women; 34 ± 4 years), ranging from recreationally active to endurance‐trained athletes, participated in this study. This study was approved by the Sterling Institutional Review Board (Atlanta, GA) for the protection of human study participants. Subjects were informed of the experimental procedures and associated risks before providing written informed consent. All trials were completed in the winter and early spring months (December through April) in northeast Illinois; therefore, subjects were not heat acclimatized.

For ecological relevance, subjects with tattoos (*n* = 4 upper arm, *n* = 1 chest, *n* = 4 upper back, and *n* = 2 calf) were included in this study. The mean age of the tattoos was 11 years and ranged from 2 months to 21 years. In addition, multiple ethnicities/races (*n* = 44 Non‐Hispanic or Latino, Caucasian; *n* = 2 Hispanic or Latino, Caucasian; *n* = 2 Non‐Hispanic or Latino, Asian; *n* = 1 Non‐Hispanic or Latino, African American) and a range of training status/aerobic fitness levels were included. In the results section, data from tattooed skin sites (*n* = 0 to 4, depending upon site), participants of ethnicities/races other than Non‐Hispanic or Latino, Caucasian (*n* = 5), and highly fit, endurance‐trained individuals (*n* = 6) are labeled individually to visually illustrate potential impact on the primary outcome measures.

### 2.2 Preliminary screening measurements

During a screening visit subjects’ nude body mass (to the nearest 0.01 kg) and height (to the nearest 0.1 cm) were measured. Subjects also completed a graded exercise test to assess for the presence of arrhythmias or cardiovascular abnormalities (12‐lead ECG, Schiller AT‐10 Plus; Schiller America) and to determine maximum heart rate (HR_max_) and VO_2peak_ (MOXUS; AEI Technologies) on a cycle ergometer (Velotron SRAM, Pro) or treadmill (HPCosmos, Cosmed T200).

### 2.3 Experimental procedures

For the experimental trials, subjects reported to the laboratory at 08:00 or 13:00 after abstaining from caffeine, alcohol, and vigorous exercise for 24 hr and food for 2 hr. Subjects were asked to swallow an ingestible temperature sensor (CorTemp®; HQ Inc.) and drink 500 ml of water 2 hr before the trials. A urine sample was collected for assessment of baseline urine specific gravity (USG; Atago Pen Refractometer, 3741‐E03.). During the experimental trials, subjects cycled on an ergometer (Velotron SRAM, Pro) for 90 min in a warm environment (32°C). Regional absorbent patches were applied to the skin 15 min after the onset of exercise and removed upon moderate sweat absorption. Subjects were assigned to the low (25%, *n* = 24) and moderate (50%, *n* = 25) relative humidity trials in a randomized, counterbalanced manner. Heart rate was monitored using telemetry (Polar Electro RS400; Lake Success, NY). The cycle ergometer was set to a power output that corresponded to 80%–85% HR_max_. Body core temperature (CorTemp® Data Recorder; HQ Inc.), ratings of perceived exertion (RPE) (Borg, 1982), power output (watts), and cadence (revolutions per min) were recorded every 10 min. Energy expenditure (kcal) was calculated from the cycling work rate (ACSM, 2014). Subjects were provided a commercially available 6% carbohydrate–electrolyte solution (at room temperature, 32°C) to drink ad libitum during exercise. The washdown technique was used to determine whole sweat electrolyte concentrations. During exercise two front‐facing (lower body 2.9–3.1 m/s, upper body 2.3–3.0 m/s) fans and one rear‐facing (1.5–2.0 m/s) fan were used to facilitate sweat evaporation and prevent sweat dripping to the floor. Immediately before exercise and immediately after the postexercise washdown procedures, nude body mass was recorded using a digital platform scale (KCC300 platform and IND439 reader; Mettler Toledo, Columbus, OH) to the nearest 0.01 kg. Subjects were asked to towel dry before each body mass measurement.

### 2.4 Regional sweat collection

Before exercise, the subject's skin was shaved and cleaned with alcohol at the patch locations. The absorbent patch technique (Tegaderm^TM^ + Pad (3M, St. Paul, MN); pad size 11.9 cm^2^ with an absorbent capacity of ~1.3–1.5 g) was used to collect sweat from the following anatomical sites on the right side of the body: calf, ventral midthigh, upper arm (triceps), upper chest, upper back (scapular region), dorsal midforearm, and ventral midforearm. Immediately before patch application the skin was wiped dry with electrolyte‐free gauze (4 × 4 in.; Thermo Fisher Scientific, Waltham, MA). Absorbent patch application began 15 min after the onset of exercise and was completed within 5 min. This timing was chosen to avoid collection of initial sweat secreted during the gradual ramp up to steady state sweating, while also allowing time for sufficient sample volume collection for all sites by the end of the 90‐min‐cycling protocol. Patches were applied to the seven anatomical sites in the same order for all subjects. After patch application, an elastic net dressing (Surgilast; Derma Sciences, Princeton, NJ) was put on the forearms when needed to ensure patches remained adhered to the skin.

Patches were removed upon moderate sweat absorption (aiming for ~0.5–0.7 g) but before saturation as determined by visual inspection. Actual mean ± *SD* sample volumes (and adherence durations) were: calf 0.51 ± 0.24 g (63.4 ± 9.5 min), thigh 0.55 ± 0.20 g (55.0 ± 14.2 min), upper arm 0.59 ± 0.25 g (55.3 ± 13.5 min), chest 0.72 ± 0.25 g (48.8 ± 13.2 min), upper back 0.66 ± 0.18 (38.2 ± 15.2 min), dorsal forearm 0.70 ± 0.22 g (41.2 ± 13.6 min), and ventral forearm 0.82 ± 0.20 g (40.9 ± 13.6 min). Upon removal, the absorbent pad was immediately separated from the Tegaderm using clean forceps and placed in an air‐tight plastic tube (Sarstedt Salivette). The pad was centrifuged at 3,000 revolutions/min and 4°C for 10 min to extract the sweat sample for subsequent analysis.

### 2.5 Whole‐body sweat electrolyte collection

The whole‐body washdown method was used to determine sweat [Na^+^], sweat chloride concentration ([Cl^‐^]), and sweat potassium concentration ([K^+^]) from the entire body (Lemon & Yarasheski, 1985). After the subject's skin was shaved and cleaned with alcohol at the patch locations prior to exercise, subjects’ whole bodies were rinsed with 5.0 liters of deionized water using a compression sprayer (model 010PEXG; Gilmour, Somerset, PA) and then dried with electrolyte‐free paper towels (Wypall L‐40; Kimberly Clark, Irving, TX). Next, subjects donned compression shorts/sport bra and a heart rate monitor that had been previously rinsed with deionized water to remove any electrolytes and air‐dried. Subjects did not wear socks or shoes during the trial. During exercise, care was taken to avoid sweat drippage. Two front (lower body 2.9–3.1 m/s, upper body 2.3–3.0 m/s) fans and one rear (1.5–2.0 m/s) fan were used to promote evaporative cooling. Subjects were given an electrolyte‐free paper towel to absorb sweat from their face, neck, front torso, and arms. Study investigators also wiped the subject's back with an electrolyte‐free paper towel to prevent dripping of sweat. The cycle ergometer seat and handlebars were covered with a plastic bag.

At the end of the 90 min of cycling exercise, the subjects stepped off the ergometer and directly into the washdown chamber that was positioned next to the cycle ergometer. The postexercise washdown chamber consisted of a bale bag (Farm Bag Film Division) inside a steel frame (64 in × 31 in × 36 in). The shorts/sport bra, heart rate monitor strap, and paper towels used to wipe the subjects’ sweat were hung to air dry. Next, the nude subject was rinsed thoroughly with deionized water (using a compression sprayer, N‐80; Tabor Tools) to ensure removal of all sweat electrolytes from the skin and hair. Five (5.0) liters of deionized water were prepared, of which a 200‐ml sample was separated into aliquots for prerinse analysis, and the remaining 4.8 liters were used for rinsing the subject. After rinsing, the subject dried off with electrolyte‐free paper towels and stepped out of the washdown chamber. The heart rate monitor and subject's shorts/sport bra, as well as all paper towels, gauze, elastic netting, Tegaderm™ part of the patches, and gloves that touched the subject during exercise were put in the bottom of the bale bag (with the postrinse deionized water). After the contents collected at the bottom of the bale bag were thoroughly mixed, a postrinse sample was collected for electrolyte analysis.

### 2.6 Sweat analysis

Regional and whole‐body wash sweat samples were analyzed in duplicate for [Na^+^], [Cl^‐^], and [K^+^] via ion chromatography (Dionex ICS‐3000). Corrections to regional sweat [Na^+^] were made according to a previous publication (Baker et al., 2018). Briefly, the background mmol/L of [Na^+^] and [Cl^‐^] in the absorbent patches were subtracted from the mmol/L value obtained from ion chromatography analysis. The following regression equations were used: background [Na^+^] = −4.377 ln (regional sweat mass in grams) + 4.300 (r^2^ = 0.98); background [Cl^‐^] = −1.602 ln (regional sweat mass in grams) + 3.586 (r^2^ = 0.86). No corrections were needed for [K^+^]. The coefficient of variation (CV) for measuring sweat electrolyte concentrations using the regional absorbent patch method is 3%–5% (Baker et al., 2018).

Recovery of electrolytes using the whole‐body washdown procedures was measured during six mock trials using a 2‐liter solution of artificial sweat ([Na^+^]: 19–78 mmol/l, [Cl^‐^]: 24–88 mmol/L [K^+^]: 2.6–4.8 mmol/L). Recovery of Na^+^, Cl^‐^, and K^+^ was 102%, 103%, and 98%, respectively, which suggests effective detection of electrolytes in the whole‐body washdown collection system. The reliability of measuring whole‐body sweat [Na^+^] using this method was determined in a subset of 11 subjects. Each subject completed two identical trials at the same time of day 3–28 days apart. Data from the first of the two trials were used in the overall analyses of this paper, while both trials were used to calculate the CV as a measure of method reliability. The CV for whole‐body sweat [Na^+^], [Cl^‐^], and [K^+^] was 8%, 10%, and 5%, respectively.

Calculations.

### 2.7 Sweat loss and sweating rate

Whole‐body sweat loss was calculated from the change in pre‐ to postexercise nude body mass, corrected for fluid intake (difference in drink bottle mass from pre‐ to postexercise to the nearest 0.01 g using a compact digital scale; Mettler Toledo PG6002‐S, Columbus, OH), respiratory water loss, and weight loss due to substrate oxidation (Cheuvront & Kenefick, 2017). Subjects did not void between the pre‐ and postexercise nude body mass measurements; thus, no correction was needed for urine or stool loss. Subjects were not allowed to expectorate. Whole‐body sweating rate (in mg cm^−2^ min^−1^) was calculated by dividing whole‐body sweat loss by the subjects’ body surface area and the duration of exercise (90 min). Body surface area was calculated from nude body mass and height using the Dubois and Dubois equation (Dubois & Dubois, 1916). Regional sweating rate (in mg cm^−2^ min^−1^) was measured gravimetrically based on the mass of sweat absorbed in the pad (to the nearest 0.001 g using an analytical balance; Mettler Toledo Balance XS204), the pad surface area, and the duration that the patch was on the skin.

### 2.8 Sweat electrolyte concentration

Whole‐body sweat [Na^+^] was determined from dilution calculations based on the measured [Na^+^] in the postrinse solution, the known volume of deionized water added to the bale bag (4.8 liters), and sweat loss (as described above). This is hereafter referred to as “measured” whole‐body sweat [Na^+^]. In this paper “raw” regional sweat [Na^+^] refers to the regional sweat [Na^+^] values measured in the present study. Regression equations from Baker et al., 2018 (Baker et al., 2018) were applied to raw regional sweat [Na^+^] to determine “predicted” whole‐body sweat [Na^+^]. The equations were as follows per site (Baker et al., 2018):

Dorsal Forearm:y=0.589x+14.299

Ventral Forearm:y=0.682x+16.839

Upper Arm:y=0.565x+12.927

Chest:y=0.521x+10.934

Upper Back:y=0.496x+7.655

Ventral Thigh:y=0.831x+11.775

Calf:y=0.773x+15.638

where y is whole‐body sweat [Na^+^] and x is regional sweat [Na^+^]. Whole‐body and raw regional sweat

[Cl^‐^] and [K^+^] were also determined according to the same methods as [Na^+^] listed above, but only descriptive data are shown. Na^+^ is the focus of this study since it has been found to be the most important electrolyte for rehydration (Shirreffs & Sawka, 2011).

### 2.9 Statistical analyses

Analyses were carried out using Statistical Analysis Software version 9.4 (SAS Institute) and Minitab 17 Statistical Software (Minitab). The significance level for all statistical tests was set at α = 0.05. Shapiro–Wilk tests were conducted to assess normality of the data. In instances of deviation from normality, data were natural‐log transformed prior to analyses. Data are shown as mean ± standard deviation. The final sample size for analysis was *n* = 49 for all anatomical sites except the upper arm. For one subject the patch on the upper arm became unadhered to the skin prematurely. Therefore, data from this patch were excluded and final *n* = 48 for the upper arm.

Simple linear regressions and Pearson product‐moment correlations were conducted to determine the relations between regional and whole‐body sweat [Na^+^]. The prediction strength of regional to whole‐body sweat [Na^+^] was assessed via coefficients of determination (*r* ^2^) of the linear regression models (Thomas & Nelson, 2001). Quantitative agreement between raw and measured and predicted and measured sweat [Na^+^] was assessed using the concordance correlation coefficient (CCC), which measures the degree of departure between x‐axis and y‐axis values relative to perfect concordance, or the line of identity (Lin, 1989). A CCC > 0.80 is considered very good agreement (Watson & Petrie, 2010).

Bland–Altman analysis of mean bias (raw and predicted minus measured) and 95% limits of agreement (LOA) were used to compare raw (uncorrected) regional sweat [Na^+^] and predicted whole‐body sweat [Na^+^] to measured whole‐body sweat [Na^+^]. Each subject served as their own control for comparison among raw regional sweat [Na^+^], measured whole‐body sweat [Na^+^], and predicted whole‐body sweat [Na^+^]. Effect sizes were also calculated for these comparisons using Cohen's d (Cohen, 1988).

One‐way ANOVA with Dunnett's post hoc was used to compare regional to whole‐body responses for sweating rate and sweat [Na^+^]. Two‐sample *t*‐tests were used to compare subject characteristics, trial data (cycling, environment, and physiological responses), sweating rate, and sweat [Na^+^] between low and moderate relative humidity groups. Two‐sample *t* tests were also used to determine if there was a difference between low and moderate relative humidity groups in the mean bias between predicted and measured whole‐body sweat [Na^+^]. In addition, relative humidity and relative humidity‐by‐regional sweat [Na^+^] interaction terms were included in regression models for each site to determine if relative humidity had a significant effect on the prediction of whole‐body sweat [Na^+^] from regional sweat [Na^+^]. This analysis was also conducted to determine if relative humidity had a significant effect on the regression models predicting whole‐body sweating rate from regional sweating rate.

## 3 RESULTS

### 3.1 Subject characteristics

Subjects body mass, height, and VO_2peak_ were 75.09 ± 12.43 kg, 176.9 ± 8.9 cm, 48.1 ± 7.9 ml kg^−1^ min^−1^, respectively. Six male subjects were highly fit, endurance‐trained athletes (VO_2peak_ 61.0 ± 3.6 ml kg^−1^ min^−1^) while the remaining 43 subjects were recreationally active or moderately trained (VO_2peak_ 46.3 ± 6.4 ml kg^−1^ min^−1^).

There were no significant differences between low and moderate relative humidity groups for body mass (74.44 ± 12.52 kg vs. 76.54 ± 13.04 kg, *p* = .73), height (178.2 ± 7.8 cm vs. 176.2 ± 10.1 cm, *p* = .33), body surface area (1.92 ± 0.19 m^2^ vs. 1.93 ± 0.21 m^2^, *p* = .92), VO_2peak_ (49.9 ± 7.6 ml kg^−1^ min^−1^ vs. 46.8 ± 8.2 ml kg^−1^ min^−1^, *p* = .19), or age (34 ± 4 years vs. 33 ± 5 years, *p* = .90).

### 3.2 Environmental, cycling, and physiological descriptive data

Air temperature and relative humidity across all trials was 32.2 ± 0.2°C and 38.5% ± 12.2%, respectively. Body core temperature at baseline was 37.0 ± 0.4°C, average across the 90 min of exercise was 38.0 ± 0.4°C, and final temperature was 38.3 ± 0.4°C. Cycling workload was 158 ± 43 watts and total energy expenditure was 957 ± 223 kcal. Relative exercise intensity was 62% ± 7% VO_2peak_ and 82% ± 5% HR_max_. Rating of perceived exertion was 13 ± 1. Baseline urine specific gravity was 1.010 ± 0.008 and the change in body mass at the end of 90 min of cycling was −0.98% ± 0.71%.

There were no significant differences between low and moderate relative humidity groups for baseline urine specific gravity (1.010 ± 0.008 vs. 1.010 ± 0.007, *p* = .84), air temperature (32.2 ± 0.2°C vs. 32.2 ± 0.2°C, *p* = .16), energy expenditure (1,017 ± 244 kcal vs. 907 ± 187 kcal, *p* = .06), rating of perceived exertion (13 ± 1 vs. 13 ± 2, *p* = .33), relative intensity (HR_max_: 82 ± 5% vs. 82 ± 5%, *p* = .87; VO_2peak_: 64 ± 7% vs. 60 ± 7%, *p* = .09), or body core temperature (baseline: 37.0 ± 0.3°C vs. 37.1 ± 0.4°C, *p* = .19; exercise: 37.9 ± 0.4°C vs. 38.1 ± 0.4°C, *p* = .62; final: 38.2 ± 0.4°C vs. 38.3 ± 0.4°C, *p* = .50). As expected, relative humidity was significantly lower during the low humidity than the moderate humidity group (26.2 ± 1.4% and 49.3 ± 4.8%, *p* < .0001). Workload (172 ± 47 watts vs. 146 ± 36 watts, *p* = .03) and body mass loss (−1.22 ± 0.54% vs. −0.75 ± 0.78%, *p* = .01) were greater in the low versus moderate humidity group.

### 3.3 Sweat electrolyte concentrations and sweating rate

Table 1 shows whole‐body and regional sweat [Na^+^] and sweating rate for all subjects combined. Sweat [Na^+^] and sweating rate were significantly higher on most regional sites compared with that of whole‐body. Whole‐body sweat [Cl^‐^] and [K^+^] were 40.4 ± 16.8 mmol/L and 4.5 ± 0.7 mmol/L, respectively. Regional sweat [Cl^‐^] and [K^+^] were as follows: dorsal forearm 47.7 ± 23.2 mmol/L and 4.6 ± 0.8 mmol/L, ventral forearm 36.9 ± 23.2 mmol/L and 4.8 ± 1.0 mmol/L, upper arm 48.5 ± 23.7 mmol/L and 4.6 ± 0.8 mmol/L, chest 60.9 ± 23.8 mmol/L and 3.5 ± 0.7 mmol/L, upper back 61.9 ± 24.9 mmol/L and 3.8 ± 0.8 mmol/L, thigh 37.3 ± 19.8 mmol/L and 4.5 ± 0.8 mmol/L, calf 33.0 ± 21.0 mmol/L and 4.9 ± 0.9 mmol/L.

**Table 1:** Whole‐body and regional sweat sodium concentration and sweating rate for all subjects combined

	Sweat [Na^+^] (mmol/L)	Sweating Rate (mg/cm^2^/min)
Whole‐body	41.1 ± 15.6	0.805 ± 0.218
Dorsal forearm	51.3 ± 21.5*	1.684 ± 1.061*
Ventral forearm	41.2 ± 21.8	1.950 ± 1.022*
Upper Arm	50.7 ± 21.7*	0.973 ± 0.549
Chest	62.5 ± 22.9*	1.364 ± 0.614*
Upper Back	64.1 ± 23.0*	1.644 ± 0.704*
Thigh	40.8 ± 18.2	0.923 ± 0.440
Calf	37.6 ± 19.6	0.720 ± 0.387

Note

*n* = 49 for all except upper arm (*n* = 48). [Na^+^], sodium concentration.

* *p* < .0001 vs. whole‐body (one‐way ANOVA with Dunnett's post hoc).

### 3.4 Cross‐validation results

Table 2 shows the CCC results with 95% confidence intervals. The CCC for predicted versus measured whole‐body sweat [Na^+^] was ≥0.80 for all sites. The CCC for raw regional sweat [Na^+^] versus measured whole‐body sweat [Na^+^] was >0.80 for the ventral forearm, thigh, and calf and <0.80 for the dorsal forearm, upper arm, chest, and scapula.

**Table 2:** Concordance correlation coefficients with 95% confidence intervals

	Predicted whole‐body sweat [Na^+^] vs. Measured whole‐body sweat [Na^+^]	Raw regional sweat [Na^+^] vs. Measured whole‐body sweat [Na^+^]
Dorsal forearm	0.85 (0.76, 0.91)	0.74 (0.63, 0.83)
Ventral forearm	0.89 (0.81, 0.93)	0.87 (0.80, 0.91)
Upper arm	0.88 (0.80, 0.92)	0.77 (0.66, 0.85)
Chest	0.80 (0.69, 0.88)	0.49 (0.35, 0.61)
Upper back	0.82 (0.72, 0.89)	0.48 (0.35, 0.59)
Thigh	0.87 (0.79, 0.92)	0.90 (0.83, 0.94)
Calf	0.86 (0.76, 0.92)	0.84 (0.75, 0.90)

Note

*n* = 49 for all except upper arm (*n* = 48); [Na^+^], sodium concentration.

**Figure 1:**
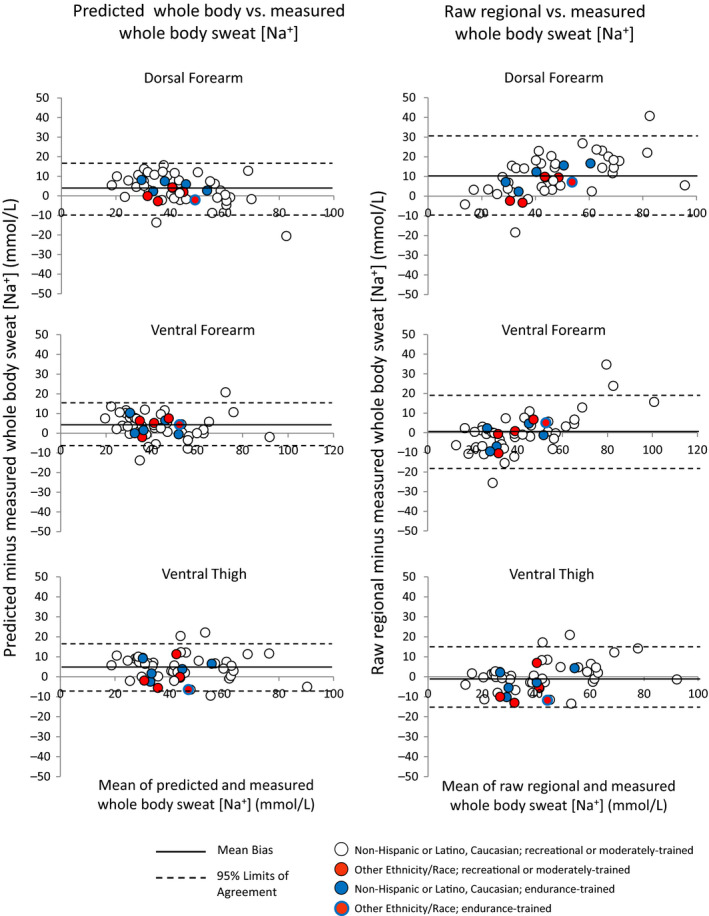
Bland–Altman figures for dorsal forearm, ventral forearm, and ventral thigh

**Figure 2:**
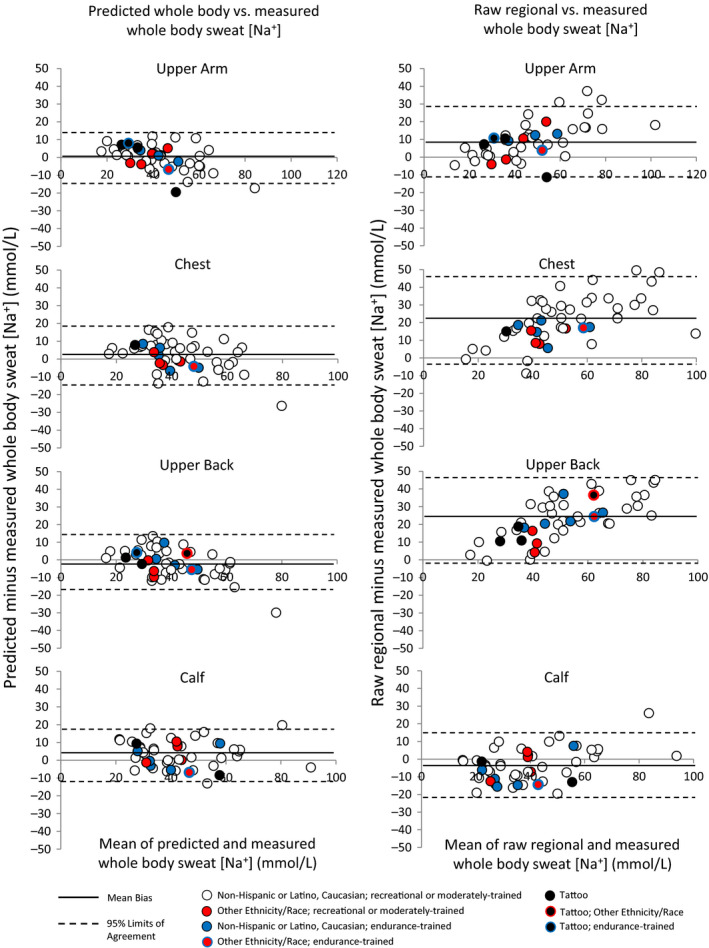
Bland–Altman figures for upper arm, chest, upper back, and calf

**Figure 3:**
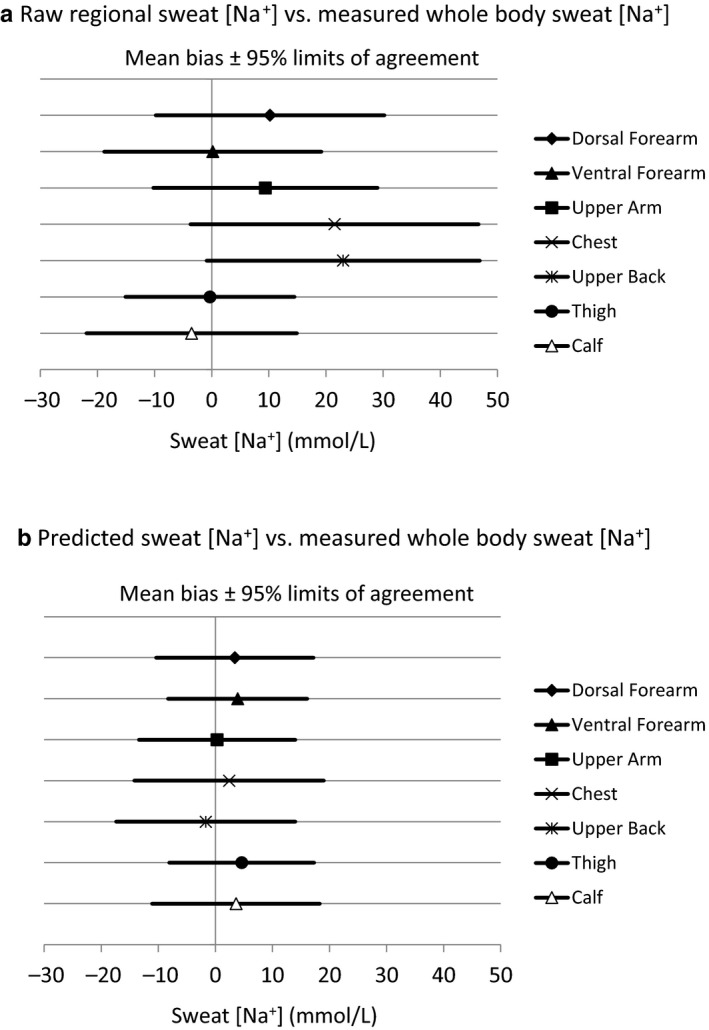
Summary of mean bias ±95% limits of agreement for all regions

Comparisons of measured whole‐body sweat [Na^+^] versus predicted whole‐body and raw regional sweat [Na^+^] are shown in Table 3. Bland–Altman figures are shown in Figures 1 and 2, with Figure 1 including sites without tattoos (dorsal forearm, ventral forearm, thigh) and Figure 2 including sites with tattoos (upper arm, chest, upper back, calf). Figure 3 shows a summary of mean bias ±95% LOA for all regions. In summary, predicted whole‐body sweat [Na^+^] from all sites was within a mean bias of 0–5 mmol/L and within a 95% LOA of ±12–17 mmol/L compared with measured whole‐body sweat [Na^+^]. The bias was generally higher for raw regional sweat [Na^+^], especially for the dorsal forearm, upper arm, chest, and upper back, where the mean difference from measured whole‐body sweat [Na^+^] was 9–23 mmol/L. The 95% LOA for raw regional sweat [Na^+^] was ±15–25 mmol/L across all sites.

**Table 3:** Comparison of measured whole‐body sweat sodium concentration versus predicted whole‐body and raw regional sweat sodium concentration

	Predicted whole‐body sweat [Na^+^] vs. Measured whole‐body sweat [Na^+^]			Raw regional sweat [Na^+^] vs. Measured whole‐body sweat [Na^+^]		
	Difference ± *SD* (mmol/L)	Effect Size	95% LOA (mmol/L)	Difference ± *SD* (mmol/L)	Effect Size	95% LOA (mmol/L)
Dorsal forearm	3.4 ± 7.1*	0.27	−10.4 to 17.2 (±13.8)	10.2 ± 10.2*	0.47	−9.8 to 30.2 (±20.0)
Ventral forearm	3.9 ± 6.2*	0.26	−8.3 to 16.1 (±12.2)	0.2 ± 9.7	0.01	−18.9 to 19.2 (±19.0)
Upper arm	0.3 ± 7.0	0.03	−13.3 to 14.0 (±13.7)	9.4 ± 10.0*	0.43	−10.1 to 29.0 (±19.6)
Chest	2.4 ± 8.5	0.20	−14.2 to 19.1 (±16.6)	21.5 ± 12.8*	0.94	−3.7 to 46.6 (±25.2)
Upper back	−1.7 ± 8.0	0.15	−17.4 to 14.1 (±15.7)	23.0 ± 12.2*	1.00	−1.0 to 46.9 (±23.9)
Thigh	4.6 ± 6.5*	0.30	−8.1 to 17.3 (±12.7)	−0.3 ± 7.5	0.02	−15.0 to 14.5 (±14.8)
Calf	3.6 ± 7.5*	0.24	−11.0 to 18.3 (±14.7)	−3.5 ± 9.4	0.18	−21.8 to 14.9 (±18.4)

Note

*n* = 49 for all except upper arm (*n* = 48). [Na^+^], sodium concentration. Effect size definitions per Cohen (Cohen, 1988): small: <0.2, moderate: 0.2– 0.8, large: >0.8.

* *p* < .05 Predicted whole‐body versus measured whole‐body or raw regional versus measured whole‐body (one‐way ANOVA with Dunnett's post hoc); difference calculated as predicted minus measured.

Based on visual inspection of Figures 1 and 2 there are no apparent effects of tattoos, ethnicity/race, or high aerobic fitness/endurance training on the results; that is, these data points do not appear to be outliers on the Bland–Altman plots comparing predicted and measured whole‐body sweat [Na^+^]. Table 4 shows individual data for predicted versus measured whole‐body sweat [Na^+^] for tattooed sites.

**Table 4:** Individual data for predicted versus measured sweat sodium concentration for tattooed sites

Tattooed Sites (*n* = 11)	Predicted whole‐body sweat [Na^+^] (mmol/L)	Measured whole‐body sweat [Na^+^] (mmol/L)	Difference (mmol/L)
Upper arm	29.9	22.9	7.0
Upper arm	36.1	30.5	5.6
Upper arm	33.4	25.4	8.0
Upper arm	40.3	59.7	−19.4
Chest	30.7	22.9	7.8
Upper back	24.2	22.9	1.3
Upper back	28.2	30.5	−2.3
Upper back	29.6	25.4	4.2
Upper back	47.5	43.8	3.7
Calf	32.2	22.9	9.3
Calf	53.5	61.9	−8.4

Note

[Na^+^], sodium concentration.

### 3.5 Effect of relative humidity on the comparison of predicted versus measured whole‐body sweat sodium concentration

Whole‐body and regional sweat [Na^+^] and sweating rate for the low relative humidity and moderate humidity groups are shown in Table 5. There were no significant differences between groups (*p* = .41–0.97). Mean bias between predicted and measured whole‐body sweat [Na^+^] was not different between low and moderate relative humidity trials for any of the regional sites: dorsal forearm 3.5 ± 6.0 mmol/L versus 3.3 ± 8.0 mmol/L (*p* = .90), ventral forearm 2.9 ± 5.0 mmol/L versus 4.8 ± 7.2 mmol/L (*p* = .28), upper arm −0.2 ± 7.8 mmol/L versus 0.8 ± 6.3 mmol/L (*p* = .64), chest 2.7 ± 7.4mmol/L versus 2.2 ± 9.6 mmol/L (*p* = .86), upper back −2.2 ± 6.6 mmol/L versus −1.0 ± 9.7 mmol/L (*p* = .64), thigh 4.6 ± 8.0 mmol/L versus 4.6 ± 4.8 mmol/L (*p* = .99), calf 3.5 ± 9.1 mmol/L versus 3.8 ± 5.6 mmol/L (*p* = .90).

**Table 5:** Whole‐body and regional sweat sodium concentration and sweating rate for each condition

	25% relative humidity		50% relative humidity	
	Sweat [Na^+^] (mmol/L)	Sweating Rate (mg/cm^2^/min)	Sweat [Na^+^] (mmol/L)	Sweating Rate (mg/cm^2^/min)
Whole‐body	42.2 ± 13.4	0.824 ± 0.220	40.1 ± 17.7	0.787 ± 0.219
Dorsal forearm	53.3 ± 20.6	1.576 ± 0.810	49.3 ± 22.6	1.788 ± 1.265
Ventral forearm	41.4 ± 19.5	1.827 ± 0.774	41.1 ± 24.3	2.069 ± 1.218
Upper Arm	52.1 ± 20.2	0.931 ± 0.593	49.4 ± 23.3	1.012 ± 0.514
Chest	65.0 ± 22.9	1.348 ± 0.604	60.2 ± 23.2	1.380 ± 0.636
Upper Back	65.1 ± 22.3	1.584 ± 0.747	63.1 ± 24.1	1.702 ± 0.671
Thigh	42.1 ± 17.6	0.951 ± 0.456	39.6 ± 19.0	0.896 ± 0.432
Calf	38.8 ± 19.2	0.736 ± 0.406	36.5 ± 20.3	0.705 ± 0.376

Note

No significant differences between 25% and 50% relative humidity (two‐sample *t* tests, *p* > .05). *n* = 25 for 50% relative humidity. *n* = 24 for 25% relative humidity for all sites except upper arm (*n* = 23). [Na^+^], sodium concentration.

**Figure 4:**
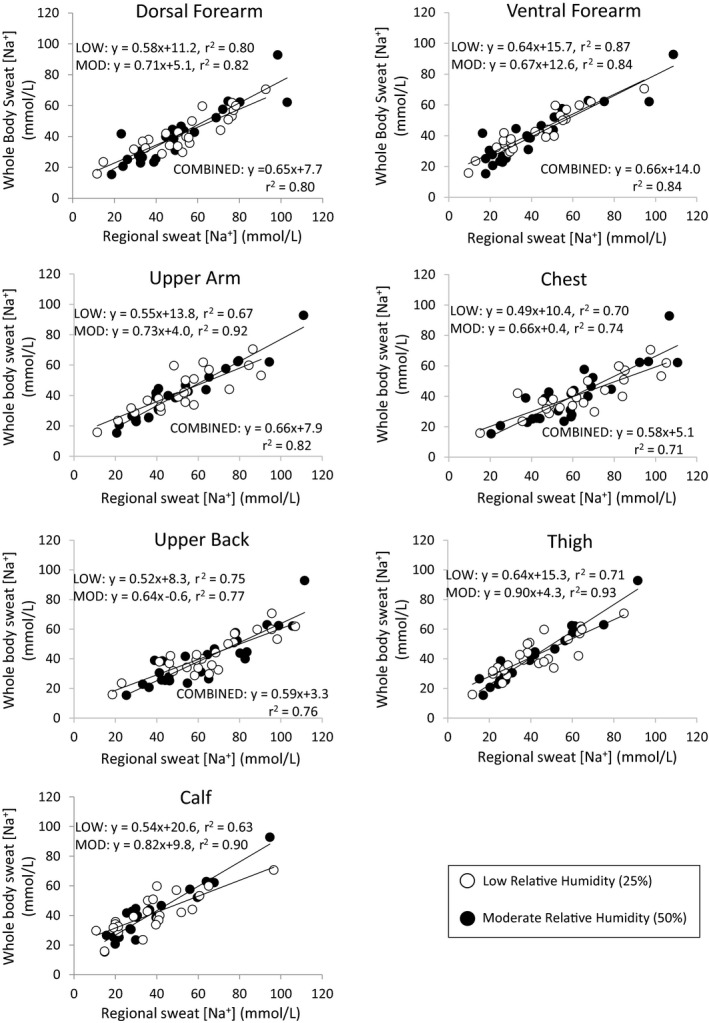
Linear regression of regional vs. whole‐body sweat sodium concentration with regression lines for low and moderate relative humidity shown separately

Figure 4 shows the scatterplots for the linear regression of regional versus whole‐body sweat [Na^+^], with regression lines shown separately for low and moderate relative humidity trials. There were no effects of relative humidity on the regression model predicting whole‐body sweat [Na^+^] from regional sweat [Na^+^] for most sites, except for the calf (*p* = .02) and thigh (*p* = .02). There were no relative humidity‐by‐regional sweat [Na^+^] interaction effects on the model for most sites, with the exception of the calf (*p* = .01) and thigh (*p* = .01). There were no effects of relative humidity (*p* = .08–0.69) or relative humidity‐by‐regional sweating rate interaction (*p* = .11–0.97) on the regression model predicting whole‐body sweating rate from regional sweating rate for any of the sites.

## 4 DISCUSSION

This study was conducted to determine the cross‐validation of published regression equations for predicting whole‐body sweat [Na^+^] from regional measures. Predicted whole‐body sweat [Na^+^] was compared with measured whole‐body sweat [Na^+^] primarily under the same experimental conditions in which the equations were developed. The main finding was that prediction equations improved the accuracy of estimating whole‐body sweat [Na^+^] over the use of raw regional sweat [Na^+^]. Predicted whole‐body sweat [Na^+^] was within a mean bias of 0–5 mmol/L and within a 95% LOA of ±12–17 mmol/L of measured whole‐body sweat [Na^+^]. Similarly, the sweat [Na^+^] prediction accuracy as assessed by the CCC indicated very good agreement between predicted and measured whole‐body sweat [Na^+^] (all sites ≥0.80).

These findings suggest that for best results published regression equations should be applied when using the regional absorbent patch method to determine an athlete's individualized sweat Na^+^ losses. This is especially important when collecting sweat from a region that produces raw sweat [Na^+^] values that are significantly higher than measured whole‐body sweat [Na^+^]. These include the dorsal forearm, upper arm, chest, and upper back, as suggested by this and previous studies (Baker et al., 2009, 2018; Patterson, Galloway, & Nimmo, 2000; Shirreffs & Maughan, 1997). At these sites raw regional sweat [Na^+^] was on average 9–23 mmol/L higher than measured whole‐body sweat [Na^+^], with 95% LOA of 20–25 mmol/L. As shown in Figure 3, using the prediction equations for these sites reduced the bias to 0–3 mmol/L and the 95% LOA to 14–17 mmol/L. For context, a 23 mmol/L overestimation of sweat [Na^+^] (upper back) for an athlete with an average sweating rate of 1.0 L/h would be equivalent to 0.53 g of Na^+^ (1.3 g NaCl) per hour of exercise. On average raw regional sweat [Na^+^] from the ventral forearm, thigh, and calf were not significantly different from measured whole‐body sweat [Na^+^]. However, it is important to note that applying the prediction equations still reduced the 95% LOA at these sites slightly, from ±15–19 to ±12–15 mmol/L (Figure 3).

A secondary aim of this study was to compare predicted versus measured whole‐body sweat [Na^+^] under environmental conditions that varied from that of the original studies. The results suggest that a change in relative humidity from 25% to 50% had no impact on the mean bias between predicted and measured whole‐body sweat [Na^+^]. Likewise, for most sites relative humidity had no impact on the regression model predicting whole‐body sweat [Na^+^] from regional sweat [Na^+^]; however, there were two exceptions: the calf and thigh (Figure 4). The physiological basis for this finding is unclear, as there were no concomitant effects of relative humidity on the relation between regional and whole‐body sweating rate. Interestingly, previous research has shown that exercise intensity also has a significant impact on the relation between regional and whole‐body sweat [Na^+^] for the calf and thigh (Baker et al., 2019). This means that regression equations specific to the workload and environmental conditions would be needed to predict whole‐body sweat [Na^+^] from the calf or thigh regions. For simplicity, it is perhaps best to avoid the calf and thigh regions when sweat testing; and instead opt for other sites (e.g., dorsal forearm, ventral forearm, upper arm, chest, and upper back), for which the regional‐to‐whole‐body relation is unaffected by intensity and relative humidity (Baker et al., 2019).

In the interest of ecological relevance and determining the generalizability of the prediction models to broader populations, we included subjects with tattoos and of varying ethnicity/race and aerobic fitness levels. Based on visual assessment of the Bland–Altman plots (Figures 1 and 2) there is no apparent impact of tattoos, ethnicity/race, or aerobic fitness on the accuracy of predicted whole‐body sweat [Na^+^]. One published study has reported that mean sweat [Na^+^] from tattooed skin was significantly higher than nontattooed skin when stimulated by pilocarpine iontophoresis (Luetkemeier et al., 2017). Higher sweat [Na^+^] on tattooed skin would lead to significant overestimations of predicted whole‐body sweat [Na^+^]. If this finding held true in the present study, then tattooed sites would be consistently above the mean bias line of the Bland–Altman plots showing the difference between predicted and measured whole‐body sweat [Na^+^]. However, as illustrated in Figure 2 (left panels), the tattooed sites (represented by black data points) were scattered above and below the mean bias line in the Bland–Altman plots. Therefore, our findings seem to be in line with recent exercise studies that found no significant effect of tattoos on sweat [Na^+^] (Beliveau et al., 2020; Rogers et al., 2019) or the accuracy of whole‐body sweat [Na^+^] prediction equations (Beliveau et al., 2020).

Previous cross‐sectional studies have produced mixed results when comparing sweat [Na^+^] among groups of various aerobic fitness levels. Some reported that trained individuals have higher regional ion reabsorption rates (Amano et al., 2017) and lower sweat [Na^+^] (Araki, Matsushita, Umeno, Tsujino, & Toda, 1981; Henkin, Sehl, & Meyer, 2010), while others have found no differences (Hamouti, Del Coso, Ortega, & Mora‐Rodriguez, 2011; Henkin et al., 2010). In addition, limited research suggests there are minimal inherent ethnic/racial differences in sweat electrolyte concentrations (Kawahata & Sakamoto, 1951; Ladell, 1948; McLean, Brown, & Black, 2016; Robinson & Robinson, 1954). However, these previous studies have focused primarily on one region of sweat collection. No studies to the authors’ knowledge have investigated the effect of endurance training or ethnicity/race on regional variation of sweat [Na^+^] or the relation between regional and whole‐body sweat [Na^+^]; as these are the key factors that would impact the applicability of the prediction equations. As shown in Figures 1 and 2, the highly fit, endurance‐trained athletes (represented by blue data points) and participants of minority ethnicities/races (represented by red data points) were scattered above and below the mean bias line in the Bland–Altman plots. Therefore, our findings seem to suggest no clear effects of endurance training or ethnicity/race on the accuracy of whole‐body sweat [Na^+^] prediction equations. However, because of the small number of subjects and the qualitative aspect of this preliminary analysis the present results should be interpreted with caution. More research is needed before any firm conclusions can be made. Although the racial/ethnic makeup of our sample was representative of the local population (~90% Caucasian, ~10% non‐Caucasian), it was not representative of the national or global population. It is also important to note that these results are applicable only to moderate intensity exercise (equivalent to ~80%–85% HR_max_ or 60%–65% VO_2peak_) in warm conditions.

## 5 CONCLUSIONS

Published prediction equations improve the accuracy of estimating whole‐body sweat [Na^+^] from regional measures, especially for the dorsal forearm, upper arm, chest, and upper back. Therefore, it is recommended that appropriate regression equations are applied when using the regional absorbent patch method to determine individualized sweat electrolyte losses. Small changes in relative humidity (25%–50%) have minimal impact on the accuracy of predicted whole‐body sweat [Na^+^]. Furthermore, preliminary data suggest no clear effects of tattoos, ethnicity/race, or endurance training on the accuracy of whole‐body sweat [Na^+^] prediction equations. However, more research is needed to determine the effect of these and other (e.g., air temperature, heat acclimation) factors on the relation between regional and whole‐body sweat [Na^+^].

## CONFLICT OF INTEREST

All authors are employed by PepsiCo R&D. The views expressed in this manuscript are those of the authors and do not necessarily reflect the position or policy of PepsiCo, Inc.

## AUTHOR CONTRIBUTIONS

LBB and KAB conceived and designed the research. KAB, RPN, AJR, and SDB conducted the experiments and CU and SDB conducted biochemistry analysis. LBB and PJD analyzed the data. LBB drafted the manuscript, and KAB, RPN, AJR, SDB, CTU, and PJD read, edited, and approved the manuscript.
